# Identification of nanoparticle infiltration in human breast milk: Chemical profiles and trajectory pathways

**DOI:** 10.1073/pnas.2500552122

**Published:** 2025-05-12

**Authors:** Qing Yang, Di Chen, Xi Liu, Wenjie Li, Huizhen Zheng, Xiaoming Cai, Ruibin Li

**Affiliations:** ^a^Center for Genetic Epidemiology and Genomics, School of Public Health, Suzhou Medical College, Soochow University, Suzhou, Jiangsu 215123, China; ^b^Collaborative Innovation Center of Radiological Medicine of Jiangsu Higher Education Institutions, Suzhou Medical College, Soochow University, Suzhou, Jiangsu 215123, China; ^c^State Key Laboratory of Radiation Medicine and Protection, School for Radiological and Interdisciplinary Sciences, Suzhou Medical College, Soochow University, Suzhou, Jiangsu 215123, China; ^d^Nanotechnology Centre, Centre for Energy and Environmental Technologies, Vysoká škola báňská-Technical University of Ostrava, Ostrava 70800, Czech Republic

**Keywords:** nanoparticle, biodistribution, human milk, pollutant

## Abstract

Breast milk is crucial for infant health, offering essential nutrients and immune protection. However, despite increasing exposure risks from nanoparticles (NPs), their potential infiltration into human breast milk remains poorly understood. This study provides a comprehensive chemical profile of NPs in human breast milk, analyzing their elemental composition, surface charge, hydrodynamic size, and crystallinity. NPs were detected in 42 out of 53 milk samples, with concentrations reaching up to 1.12 × 10^11^ particles/mL. These particles comprised nine elements, with O, Si, Fe, Cu, and Al being the most frequently detected across all samples. We establish a mechanistic axis for NP infiltration, involving penetration of the intestine/air–blood barriers, circulation in blood vessels, crossing the blood–milk barrier via transcytosis or immune cell-mediated transfer, and eventual accumulation in milk. Structure–activity relationship analysis reveals that smaller, neutral-charged NPs exhibit stronger infiltration capacity, offering potential for regulating NP behavior at biological barriers through engineering design. This study provides the chemical profiles of NPs in human breast milk and uncovers their infiltration pathways.

## Significance

Despite rising exposure risks, the potential impacts of nanoparticles (NPs) on human breast milk remain largely unexplored. This study provides a comprehensive chemical profile of NP contaminants in human breast milk. It establishes a mechanistic framework linking maternal NP exposure to infiltration in breast milk, detailing barrier penetration, circulation, and transfer pathways. Furthermore, it offers critical insights into structure–activity relationships (SAR), highlighting how NP size and surface charge influence their ability to cross biological barriers.

Breast milk is valuable for infant health, offering critical nutrients and immune protection that support growth and development (1–4). However, maternal exposure to environmental pollutants during pregnancy or lactation can lead to breast milk contamination, potentially affecting infant health (5–8). Over recent decades, significant efforts have focused on protecting maternal health from hazardous substances, such as heavy metals (9–11) (e.g., lead, mercury, cadmium), environmentally persistent organic pollutants (12, 13) (e.g., dioxins, polychlorinated biphenyls, polyfluoroalkyl substances), and pathogens (14, 15) (e.g., HIV, hepatitis B virus). These hazardous agents can breach biological barriers, contaminate breast milk, and pose serious risks to infants (6, 8, 16). Therefore, it is important to ensure the quality of breast milk by managing these pollutants. Regulatory agencies have established guidelines to minimize maternal exposure to harmful contaminants. For example, the US Food and Drug Administration (FDA) and the US Environmental Protection Agency (EPA) jointly advise pregnant and breastfeeding women to limit mercury exposure by avoiding high-mercury seafood such as swordfish, marlin, and tuna (17). Research has also linked maternal habits to polychlorinated biphenyl (PCB) levels in human milk, suggesting that breastfeeding women should limit consumption of fish from unknown origins and avoid tobacco smoking to reduce PCB exposure (18). Additionally, the World Health Organization (WHO) advises that mothers living with HIV should receive antiretroviral therapy during breastfeeding to reduce postnatal transmission risks to infants (19). In addition to these well-recognized toxins, growing attentions are being drawn on a new category of fine particulate matter: nanoparticles (NPs) such as SiO_2_, TiO_2_, ZnO, and Ag (20–22). With the rapid development of nanotechnology, NPs are increasingly released into the environment, contaminating ambient air, water, and food sources (23, 24). Once entering the human body, NPs can interact with biological systems and potentially induce adverse outcomes, including lung fibrosis, carcinogenesis, inflammation, and immune suppression (25–27). Despite the increasing health risks from NPs, their potential infiltration into human breast milk remains largely unexamined.

Mammals have developed sophisticated barriers to regulate the transfer of substances into breast milk. When exposed to substances through ingestion or inhalation, these agents must first penetrate the intestine–blood or gas–blood barriers to enter the systemic circulation (28, 29). Within mammary capillaries, epithelial cells selectively absorb nutrients needed for the synthesis of milk components such as proteins, fats, and lactose (30–32). These components are then secreted into the alveolar lumen through processes like exocytosis and fat globule secretion. The blood–milk barrier (BMB) acts as a critical safeguard, preventing the uncontrolled exchange of substances between the bloodstream and milk (33). However, some harmful agents can bypass this barrier. For instance, HIV enters breast milk through the migration of infected cells from the bloodstream (34), while allergenic proteins can traverse the BMB via transcytosis, involving binding, intracellular transport, and release into milk (35). NPs such as Ag (22, 36, 37), ZnO (38–40), and TiO_2_ (41) have also been reported to accumulate in mammary glands, likely via epithelial disruption. However, in vivo real-time visualization of NP transport across the BMB and experimental validation in biological models remain lacking, leaving this mechanism largely unverified. Given that the size range of NPs (1 to 100 nm) overlaps with that of proteins and viruses (42), we hypothesize that NPs may infiltrate the BMB through other mechanisms beyond epithelial barrier disruption.

To test this hypothesis, we collected milk samples from 53 breastfeeding mothers and employed a digestion-ultracentrifugation method to isolate NPs. Notably, NPs were identified in 42 milk samples. We conducted comprehensive physicochemical analyses, including assessments of composition, size, crystallinity, and surface charge, to establish a detailed chemical profile of these NPs. To further understand their biological behavior, we focused on SiO_2_‚ NPs, a representative particle frequently detected in the milk samples, and investigated their interactions at the blood–milk barrier in a mouse model following oral and respiratory exposure. SiO_2_ NPs were labeled with metallic (gold NPs) and fluorescent (fluorescein isothiocyanate, FITC) tags, generating FITC-SiO_2_@Au core-shell structures, enabling high-resolution tracking of their biodistribution using multimodal imaging techniques. Additionally, capillary electrophoresis coupled with laser-induced fluorescence (CE-LIF) was employed to quantify SiO_2_ NP levels in milk samples. Taken together, this study provides a comprehensive chemical profile of NPs in human breast milk and elucidates their infiltration pathways.

## Results

### Identification of NPs in Human Breast Milk.

Breast milk samples were collected from 53 nursing mothers between 1 and 11 mo postpartum (*SI Appendix*, Table S1). Nanoparticles (NPs) were isolated from the milk using a digestion-ultracentrifugation method, which involves centrifugation to collect particle pellets, followed by enzymatic digestion and thorough washing to remove biological debris. Both environmental blank (EBL) and procedural blank (PBL) samples were included for comparison.

**Fig. 1.**
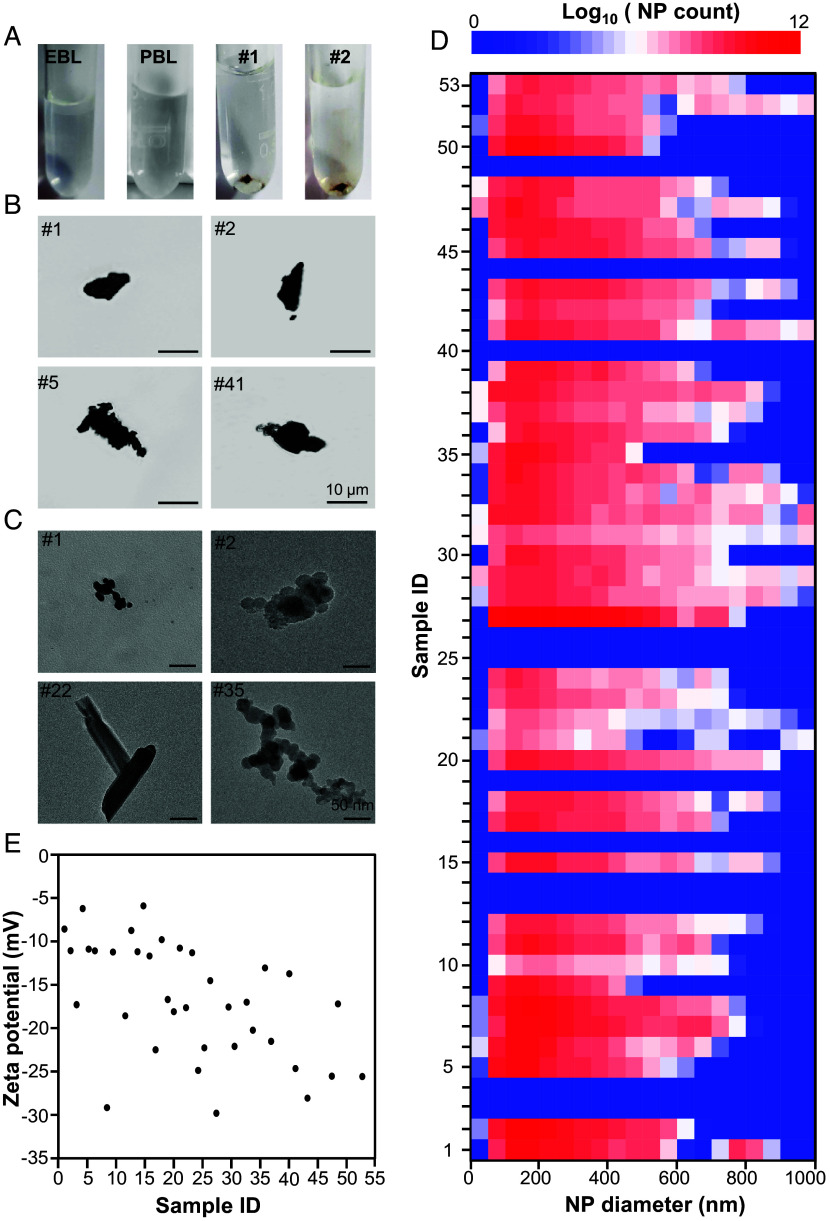
Identification of NPs from human breast milk samples. (*A*) Photographs, (*B*) optical microscopy images, and (*C*) TEM images of NP pellets isolated from representative milk samples. (*D*) Counts and size distribution of NPs in each milk sample as measured by NTA. (*E*) Zeta potential of isolated NPs in DI water.

The isolated particles were comprehensively characterized using transmission electron microscopy (TEM), light microscopy, nanoparticle tracking analysis (NTA), energy-dispersive X-ray spectroscopy (EDX), X-ray diffraction (XRD), zeta potential analysis, and inductively coupled plasma mass spectrometry (ICP-MS). In some samples, isolated NP pellets were visible to the naked eye, displaying a dark brown color (Fig. 1*A*), while most NP pellets were distinguishable under the light microscope (Fig. 1*B*). TEM images of particles from representative samples revealed spherical or lamellar shapes within the nanoscale range (Fig. 1*C* and *SI Appendix*, Fig. S1). Remarkably, NPs were detected in 42 out of 53 breast milk samples by NTA (Table 1), with no particles observed in the EBL and PBL samples. The total NP counts in 42 milk samples ranged from 6.94 × 10^6^ to 1.12 × 10^11^ particles/mL (Table 1), with an average hydrodynamic size range of 145 to 268 nm, a minimum size of 26.5 nm, and a maximum size of 1,000 nm (Fig. 1*D*). All 42 NP samples exhibited a negative surface charge, with ζ values between −30 and −5 mV (Fig. 1*E*).

**Table 1.** NP counts in human breast milk samples

Sample ID	Count (NPs/mL)	Sample ID	Count (NPs/mL)
1	2.30 × 10^10^	28	7.30 × 10^9^
2	3.98 × 10^10^	29	1.89 × 10^9^
3	ND	30	4.40 × 10^9^
4	ND	31	2.28 × 10^8^
5	2.36 × 10^10^	32	1.09 × 10^9^
6	2.58 × 10^10^	33	3.53 × 10^9^
7	2.92 × 10^10^	34	1.44 × 10^9^
8	3.66 × 10^10^	35	2.42 × 10^9^
9	9.48 × 10^9^	36	2.58 × 10^9^
10	6.94 × 10^6^	37	1.35 × 10^9^
11	1.07 × 10^10^	38	3.98 × 10^10^
12	1.09 × 10^9^	39	4.70 × 10^9^
13	ND	40	ND
14	ND	41	9.80 × 10^9^
15	4.12 × 10^10^	42	1.01 × 10^9^
16	ND	43	2.72 × 10^9^
17	3.82 × 10^9^	44	ND
18	6.05 × 10^9^	45	1.09 × 10^9^
19	ND	46	1.23 × 10^9^
20	9.10 × 10^9^	47	3.10 × 10^9^
21	1.07 × 10^10^	48	1.03 × 10^9^
22	2.14 × 10^8^	49	ND
23	1.72 × 10^8^	50	2.06 × 10^10^
24	2.57 × 10^8^	51	4.12 × 10^10^
25	ND	52	1.14 × 10^9^
26	ND	53	1.28 × 10^9^
27	1.12 × 10^11^	EBL/PBL	ND

Note: ND indicates not detectable; EBL and PBL represent environmental and PBLs, respectively.

**Fig. 2.**
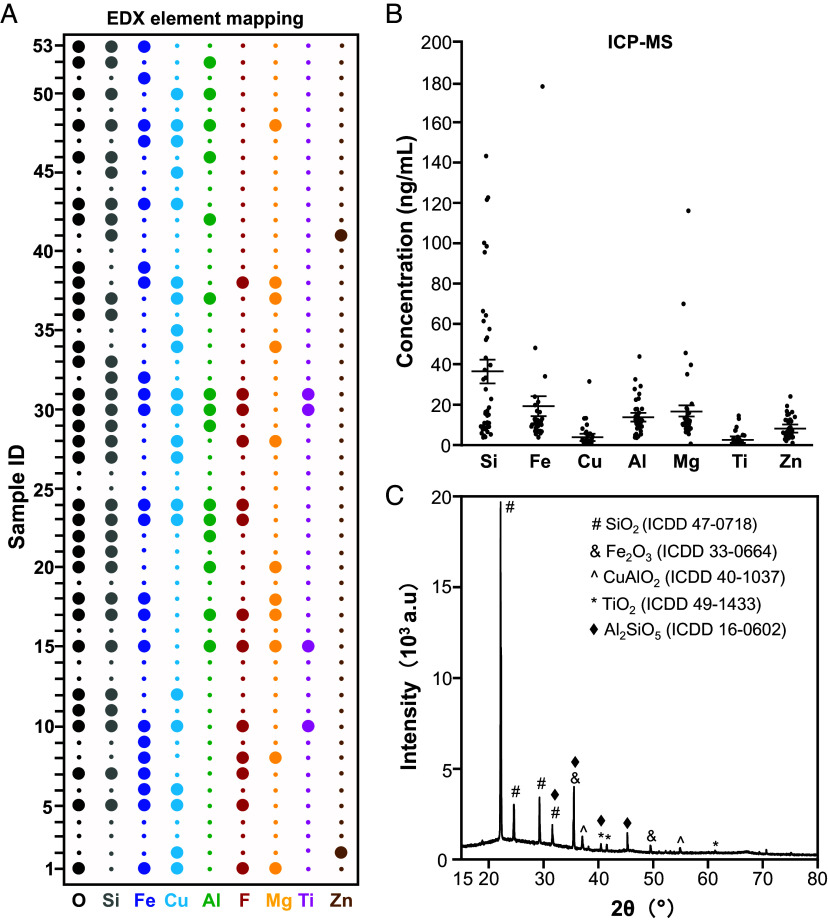
Composition analysis of NP pellets. (*A*) Elemental composition identified by EDX. Large dots indicate the detected elements. (*B*) Quantitative elemental analysis by ICP-MS. (*C*) Crystallinity analysis conducted by XRD. For XRD, NP pellets were isolated from pooled 10 mL aliquots of human breast milk samples and extensively washed before analysis.

The chemical composition of the particle pellets was examined by EDX, ICP-MS, and XRD. EDX analysis detected 9 elements in the 42 samples, including O, Si, Fe, Cu, Al, F, Mg, Ti, and Zn (Fig. 2*A*). The percentages of the metal and Si elements in each NP sample were quantified by ICP-MS analysis. As shown in Fig. 2*B* and *SI Appendix*, Table S2, Si was the most abundant element, with a mean concentration of 35.42 ng/mL, followed by 16.27 ng/mL Mg,15.80 ng/mL Fe, 13.09 ng/mL Al, 7.74 ng/mL Zn, 3.39 ng/mL Cu, and 2.25 ng/mL Ti in breast milk. A combined sample of particle pellets from the 42 milk samples was subjected to XRD analysis, which identified SiO_2_, Fe_2_O_3_, CuAlO_2_, TiO_2_, and Al_2_SiO_5_ as the principal phases of the NP pellets (Fig. 2*C*). Collectively, these findings provide robust evidence of NPs in human breast milk and offer a comprehensive chemical profile of the isolated NPs.

To assess the possible sources of NPs in breast milk, we conducted a multivariable linear regression analysis evaluating the relationship between various potential influencing factors and NP levels in human breast milk (*SI Appendix*, Table S3). We assessed 11 factors, including maternal use of products that may contain NPs (e.g., household sprays, sunscreen, makeup), dietary intake of foods that may contain NPs, and airborne particulate levels in the residential environment. Notably, our analysis identified flour ingestion as a significant contributor to NP presence in breast milk.

### Examining NP Accumulation in Breast Milk Following Oral/Respiratory Exposure.

**Fig. 3.**
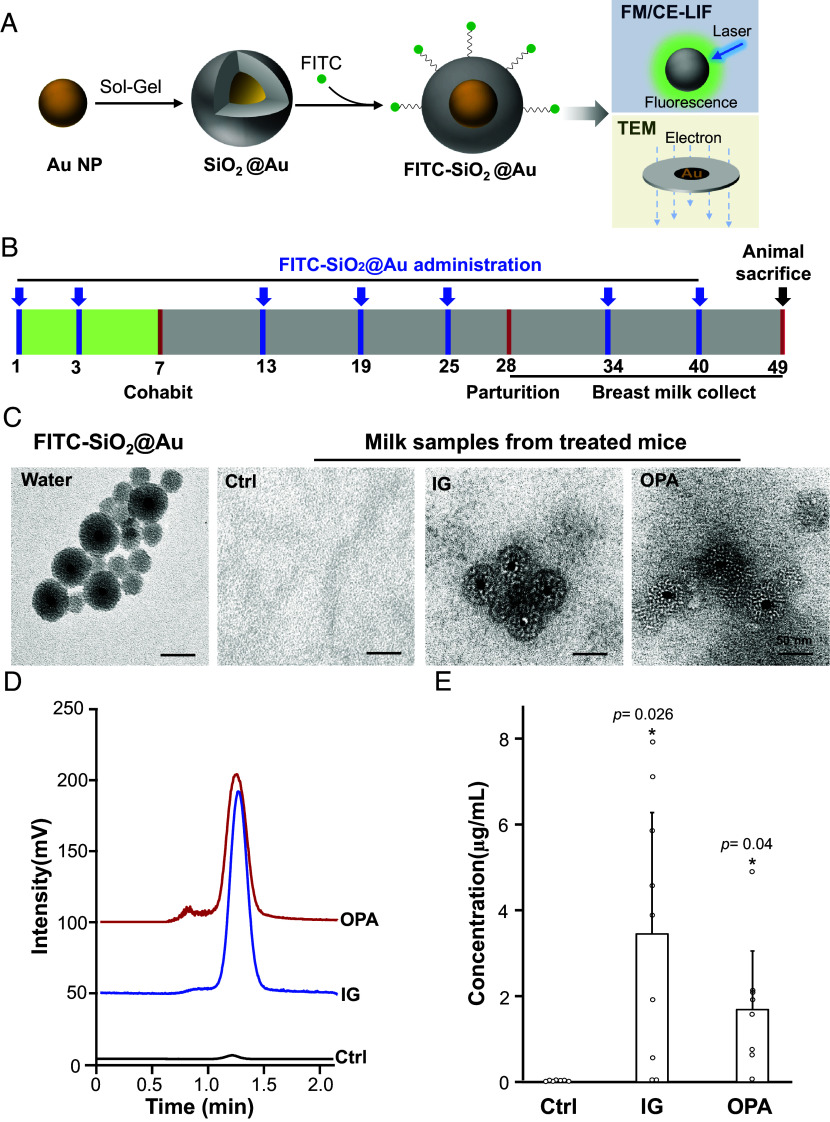
Identification of SiO_2_ NPs in mouse breast milk after oral/respiratory exposures. (*A*) Schematic illustrating metallic and fluorescent labeling methods used to identify SiO_2_ NPs. (*B*) Diagram of animal treatment procedures. (*C*) TEM images of SiO_2_@Au in mouse breast milk, with NPs isolated from milk samples of lactating mice exposed to vehicle control (n = 7) or FITC-SiO_2_@Au by OPA (n = 8) or IG (n = 9) administration. Pristine FITC-SiO_2_@Au NPs in DI water were included for comparison. (*D*) CE-LIF chromatograms and (*E*) quantification of FITC-SiO_2_@Au NPs in mouse breast milk. FITC-SiO_2_@Au NPs was quantified by peak area relative to standard curves. Data are presented as mean ± SD. *P < 0.05 compared to Ctrl by one-way ANOVA.

To determine the uptake source of NPs in breast milk, we focused on engineered SiO_2_ NPs, given their prevalence as the most abundant constituents in combined NP pellets. Due to the low electron density contrast and weak luminescence of SiO_2_ NPs, distinguishing them from biological contexts posed a challenge. To overcome this, we employed dual-labeling techniques using metallic (Au NP) and fluorophore (FITC) tags to create FITC-SiO_2_@Au, a dual-imageable NP with TEM-detectable SiO_2_@Au core-shell structures and surface fluorophores for fluorescence microscopy (FM) and CE-LIF detection (Fig. 3*A*). Female ICR mice were exposed to these labeled SiO_2_ particles via intragastric (IG) administration and oropharyngeal aspiration (OPA), as illustrated in Fig. 3*B*. Mice underwent seven rounds of NP exposure before copulation, during pregnancy, and throughout lactation, with vehicle controls for comparison. Milk samples were collected daily from lactating mice, yielding 200 to 350 µL per mouse by the end of lactation.

To confirm whether ingested or inhaled SiO_2_ NPs could cross biological barriers and accumulate in breast milk, collected milk samples were centrifuged to isolate pellets for TEM observation. Remarkably, TEM images clearly showed the FITC-SiO_2_@Au core-shell structures in milk samples from treated mice (Fig. 3*C*). Overall, the core-shell FITC-SiO_2_@Au were detected in 7 out of 9 IG-exposed and 6 out of 8 OPA-exposed mouse milk samples. Additionally, CE-LIF analysis quantified FITC-SiO_2_@Au concentrations in milk samples, with a prominent peak at 1.2 min and a detection limit as low as 10 ng/mL (*SI Appendix*, Fig. S2). The FITC-SiO_2_@Au peak area displayed excellent linearity (r^2^ = 0.99) with concentration, facilitating precise quantification of SiO_2_ particles in mouse milk. Notably, FITC-SiO_2_@Au peak intensity in milk samples from IG-exposed mice was higher than the value in OPA-exposed mice (Fig. 3*D*). CE-LIF quantification across all milk samples revealed that IG exposure resulted in a twofold higher average SiO_2_ level in breast milk compared to OPA exposure (Fig. 3*E*), suggesting that ingestion leads to more NP infiltrations into breast milk under the same exposure dose.

To explore the physicochemical properties influencing NP infiltration into breast milk, we prepared five SiO_2_ nanoforms with differing sizes and surface charges for structure–activity relationship (SAR) analysis. All five SiO_2_ NPs were labeled by FITC for fluorescent detection. As shown in *SI Appendix*, Table S4, SiO_2_-sa, SiO_2_-ma, and SiO_2_-la had similar surface charges (ζ potential between −34.8 and −29 mV) but varied in diameters (20 nm, 50 nm, and 100 nm, respectively). SiO_2_-sc and SiO_2_-sn represented small-sized SiO_2_ NPs with cationic and neutral surface charges, respectively. These NPs were administered to mice via ingestion, and breast milk samples were collected for SiO_2_ quantification by CE-LIF. Interestingly, the small-sized SiO_2_ with a neutral surface charge (SiO_2_-sn) demonstrated the highest accumulation in breast milk, whereas the medium-sized (SiO_2_-ma) and large-sized (SiO_2_-la) particle treatment showed lower infiltration levels (*SI Appendix*, Fig. S3).

### Elucidating the Transport Pathway of Ingested SiO_2_ NPs to Breast Milk.

**Fig. 4.**
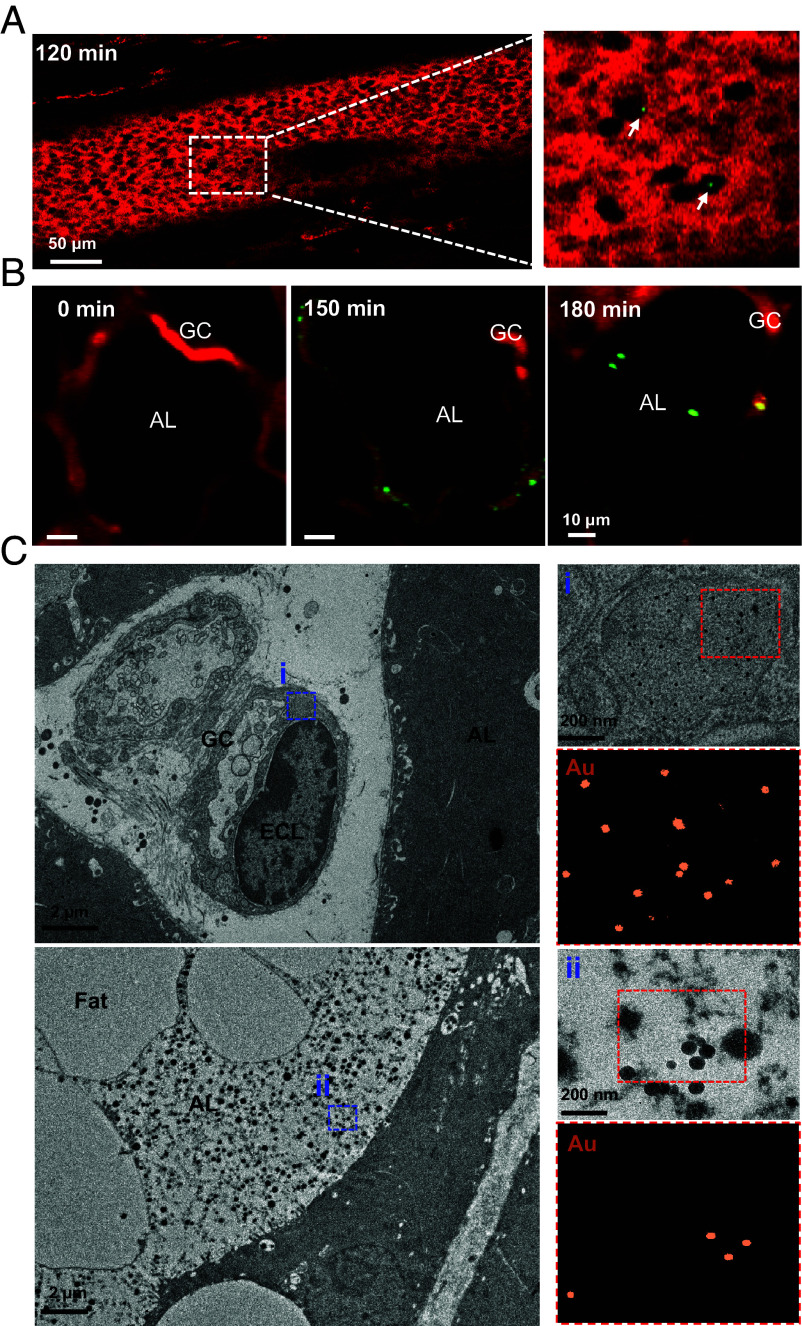
Visualization of SiO_2_ NPs in mouse mammary gland. (*A*) Representative images showing FITC-SiO_2_ NP within blood vessels and (*B*) distribution of FITC-SiO_2_ NP in mammary gland tissue. Blood vessels and mammary glands were visualized using intravital microscopy at various time points after FITC-SiO_2_ NP ingestion. Arrows indicate FITC-SiO_2_ NPs. (*C*) TEM images of SiO_2_@Au NP within the mammary gland collected at 12 h postingestion of 14 mg/kg SiO_2_@Au NP in lactating mice. Orange dots in the *Lower Right* panels indicate Au signals detected by EDX. BV: blood vessel, GC: gland capillary, ECL: epithelial cell layer, and AL: alveolar lumen.

To investigate the pathway linking environmental exposure to NP accumulation in breast milk, we focused on ingested SiO_2_ NPs to decipher the involved infiltration pathways in mouse breast milk. First, we examined the dynamics of ingested FITC-SiO_2_@Au in mouse mammary glands using intravital confocal FM. As shown in Fig. 4*A* and Movie S1, we captured the transit of SiO_2_ NPs in blood vessels (BVs) at 120 min postingestion, indicating that SiO_2_ NPs can breach the intestine–blood barrier and circulate within BVs. Next, we visualized the mammary gland area, including the gland capillary (GC), epithelial cell layer (ECL), and alveolar lumen (AL) where milk is stored. FITC-SiO_2_@Au was detected in the mammary GC at 150 min and gradually accumulated in the AL (Fig. 4*B* and Movie S2).

Furthermore, mammary glands from mice exposed to FITC-SiO_2_@Au NPs were collected and fixed for TEM visualization. Notably, we observed a typical transcytosis process of NPs at the ECL, where a significant number of FITC-SiO_2_@Au NPs were detected within vesicles of epithelial cells (panel *i* in Fig. 4*C*), displaying characteristic Au signals in the EDX analysis. Additionally, the core-shell structures of FITC-SiO_2_@Au could be detected in the AL (panel *ii* in Fig. 4*C*), suggesting the infiltration of SiO_2_ NPs into breast milk. These findings are consistent with confocal FM observations, confirming the key presentation sites of ingested SiO_2_ NPs, including BVs, GCs, ECLs, and ALs.

We next aimed to decipher the mechanisms responsible for SiO_2_ NPs penetration of the blood–milk barrier. The notable compartmentalization of SiO_2_ NPs within cytoplasmic vesicles of epithelial cells (panel *i* in Fig. 4*C*) suggests that transcytosis may be a potential pathway. Additionally, we observed an association of SiO_2_ NPs with immune cells in the BVs at 120 min postexposure (Movie S1), indicating that immune cells might facilitate SiO_2_ NP transfer across the blood–milk barrier.

**Fig. 5.**
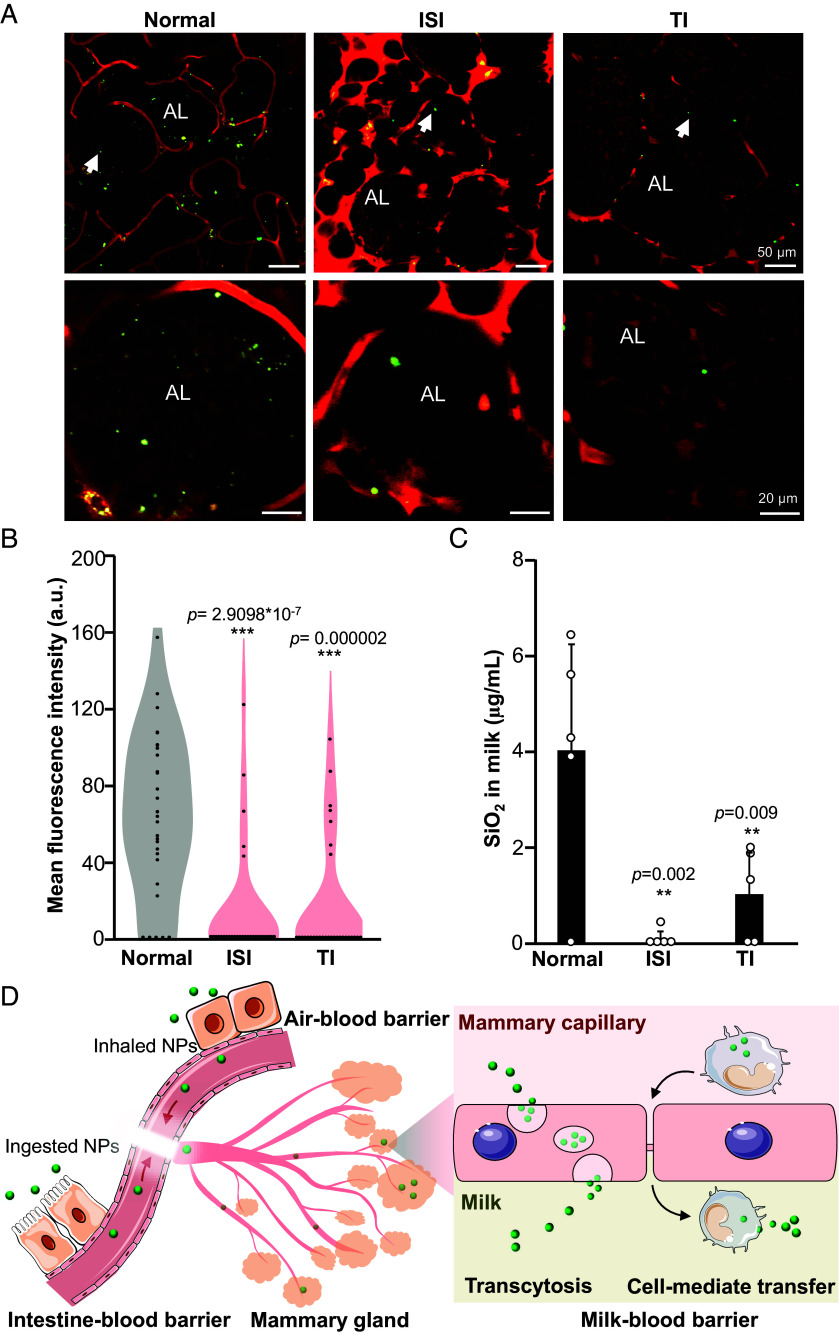
Trajectory pathway analysis of ingested SiO_2_ NPs in mouse models. (*A*) Representative microscopy images and (*B*) quantification results showing the distribution of SiO_2_ NPs in mammary glands of Ctrl mice and mice with ISI, TI following IG administration of 14 mg/kg FITC-SiO_2_. Mammary glands were visualized with an intravital microscopy at 180 min posttreatment. *Lower* panels in (*A*) show the enlarged representative ALs indicated by arrows in the *Upper* panels. Each dot in (*B*) represents the mean fluorescence intensity within single AL. (*C*) Quantification of SiO_2_ NPs in milk samples from different animal models (n = 5 for each model) by CE-LIF. Data are presented as mean ± SD. **P < 0.01, and ***P < 0.001 compared to Ctrl by one-way ANOVA. (*D*) Schematic depicting the trajectory pathway of ingested or inhaled SiO_2_ NPs, including transit across the intestine–blood or air-blood barrier, circulation in blood vessels, crossing the blood–milk barrier via transcytosis or immune cell-mediated transfer, accumulation in ALs, and eventual filtration into milk. A few cell and tissue elements were adapted and modified from figures provided by Servier Medical Art, which is licensed under CC BY 4.0 (https://creativecommons.org/licenses/by/4.0/).

To test these mechanisms, FITC-SiO_2_@Au NPs were exposed to three groups of animals: Ctrl mice, mice with immune system inhibition (ISI), and mice with transcytosis inhibition (TI). Milk samples were collected to quantify SiO_2_ levels, and mammary glands were visualized using FM to evaluate the distribution of SiO_2_ NPs. As shown in Fig. 5 *A* and *B*, significantly reduced SiO_2_ NP accumulation was observed in the ALs of ISI and TI mice, while substantial SiO_2_ NP presence was detected in the ALs of control mice. Quantitative analysis revealed that immune cell inhibition and TI resulted in 98% and 75% declines of SiO_2_ NP levels in mouse milk, respectively (Fig. 5*C*). These findings suggest that both immune cell-mediated transfer and transcytosis play critical roles in facilitating SiO_2_ NP transport across the blood–milk barrier. Based on these results, we propose a trajectory pathway for SiO_2_ NP infiltration into breast milk, involving passage through the intestine/air–blood, circulation within BVs, crossing the BMB via transcytosis or immune cell-mediated transfer, accumulation in ALs, and eventual filtration into the milk (Fig. 5*D*).

## Discussion

Extensive research has been dedicated to understanding the biological fate of NPs in vivo, with compelling evidence showing their ability to traverse biological barriers and distribute throughout various human organs and tissues. For instance, SiO_2_ and TiO_2_ nanoparticles have been detected in human liver, spleen, kidney, and intestinal postmortem samples (43, 44). Research by Qi et al. revealed that inhaled NPs could even breach both the air–blood and blood–brain barriers, making their way into the brain (45). Similarly, studies conducted by Bové et al. demonstrated that ambient black carbon particles could permeate the placental barrier, accumulating in the fetus (46). Furthermore, carbon nanotubes, which were discovered in the lungs of asthmatic patients (47) and associated with potential carcinogenic risks (48, 49), have been found to cross the air–blood barrier and travel to the liver and spleen through the bloodstream (50). Recognizing the significant threats of NP exposure to human health, the WHO has established a safety threshold (2 × 10^4^/cm^3^) for airborne NPs (<10 nm) since 2021 (51). It is noteworthy that environmental pollutants usually pose greater risks to infants compared to adults due to the immaturity of their internal organs and nervous systems. Given the high recommendation for breastfeeding to enhance infant health, the biological behavior of nanoparticles at the blood–milk barrier remains a critical but underexplored area.

In this study, we provide a comprehensive chemical profile of NPs in human breast milk. The isolated NP pellets from human breast milk contain nine elements, including O, Si, Fe, Cu, Al, F, Mg, Ti, and Zn, demonstrating the diversity of NP contaminants. We extensively analyzed the elemental composition, surface charge, hydrodynamic size, particle count, and crystal phases of the isolated NP pellets, offering a more detailed picture of NP contamination in breast milk than previous studies. XRD analysis identified five distinct crystalline structures: SiO_2_, Fe_2_O_3_, CuAlO_2_, TiO_2_, and Al_2_SiO_5_, covering most of the elements detected by EDX. However, F, Zn, and Mg were not detected in the XRD spectra, likely due to their presence in amorphous phases, or their crystalline forms being below the detection threshold of XRD.

Previous studies have reported the presence of nanoparticles in human breast milk, primarily focusing on specific types of particles, such as micro-/nanoplastics (52), carbon black (53), and metal-containing mixture particles (54), providing limited chemical profiles. For instance, micro-/nanoplastic particles (2 to 12 μm) were identified in 26 out of 34 breast milk samples, with detected constituents including polyethylene, polyvinyl chloride, and polypropylene (52). Carbon black particles were found in all eight analyzed samples, with concentrations ranging from 3.4 × 10^5^ to 1.6 × 10^6^ particles/mL (53). Additionally, Gatti et al. were the first to identify inorganic metallic nanoparticles from 19 breast milk samples (54). Compared to these works, our study provides a more comprehensive chemical profile of NPs in human breast milk, incorporating particle number, elemental composition, size distribution, surface charge, and crystal structure. This level of detail allows for a deeper understanding of the physicochemical properties of NPs in breast milk and their potential implications for maternal and infant health.

Regarding the mechanisms by which NPs infiltrate breast milk, junction gaps (38) and mammary immune cells (21, 55) have been suspected to play significant roles in facilitating NP transfer across the blood–milk barrier. Our study develops highly sensitive in vivo detection techniques, including fluorescent and metallic labeling, alongside quantification via CE-LIF. By employing Au NPs and fluorophore in FITC-SiO_2_@Au as distinct markers, we were able to accurately track NP movement across biological barriers, identifying their presence at key checkpoints such as BVs, mammary GCs, ECLs, and ALs. This approach allowed us to create a high-resolution map of the entire infiltration process from oral/respiratory exposure to milk accumulation. Notably, our study highlights transcytosis as a critical mechanism for NP transfer across the BMB, offering insights into the complexity of nanoparticle transport. We provide an in vivo video of fluorescently labeled nanoparticles traversing the BMB, enabling real-time visualization of this process. Moreover, our study is more relevant to real-world human exposure scenarios. SiO_2_ NPs are among the most widely produced and utilized NPs globally, and we employed oral and respiratory administration, which are primary environmental exposure routes, unlike prior studies that primarily relied on intravenous injections.

Although all NPs collected from human breast milk exhibited a negative surface charge, SiO_2_ NPs in mouse experiments demonstrated that neutral and positively charged NPs had stronger infiltration into milk. This discrepancy is likely due to biocorona formation. In human milk, proteins, lipids, and other small biomolecules readily adsorb onto NP surfaces, altering their physicochemical properties and shifting their charge toward negative, regardless of their original charge. In contrast, the SiO_2_ NPs used in mouse experiments were bare particles without biocorona, reflecting their intrinsic surface charge and highlighting the role of native surface properties in NP infiltration into breast milk.

The integration of human and animal studies in this work enhances our mechanistic understanding of nanoparticle (NP) infiltration into breast milk. The identification of SiO_2_ as the most abundant NP in human milk provided a strong rationale for selecting it as a model NP for trajectory pathway analysis in mice, ensuring that our animal study closely mirrors real-world human exposure scenarios. Furthermore, multivariable linear regression analysis revealed that particle-containing flour ingestion was a significant contributor to NP infiltration into human breast milk, highlighting dietary intake as a critical exposure route. This finding was further reinforced by our animal study, which demonstrated that oral exposure leads to higher NP accumulation in milk compared to respiratory exposure. The convergence of human and animal data strengthens the understanding of NP transfer mechanisms across biological barriers and underscores the importance of dietary regulation in minimizing maternal NP exposure. Reducing dietary intake of NP-contaminated foods may serve as a practical approach to minimize NP exposure to human breast milk.

## Conclusions

This study provides a comprehensive investigation into the presence, chemical profiling, and infiltration mechanisms of NPs in human breast milk. Our findings confirm the widespread presence of NPs in breast milk, with isolated NP pellets containing at least nine elements, including O, Si, Fe, Cu, Al, F, Mg, Ti, and Zn. We present a detailed chemical profile of NP contaminants in human breast milk, including chemical composition, surface charge, hydrodynamic size, particle count, and crystal phases. Using multimodal imaging techniques, we achieved high-resolution visualization of NP infiltration trajectories at critical checkpoints, including BVs, mammary GCs, ECLs, and ALs. Importantly, we identified transcytosis and immune cell-mediated transfer as key mechanisms for NP penetration across the BMB. SAR analysis further revealed that smaller, neutral-charged NPs exhibit greater infiltration into breast milk.

## Methods

### Ethics Statement.

All animal experiments were performed in accordance with the guidelines approved by the Animal Care Committee of the Laboratory Animals at Soochow University (No. 202210A0541). The research protocol on human breast milk collection and treatment was approved by the Medical Ethics Committee of Soochow University (SUDA20241209H03).

### Chemicals and Materials.

Urea (Reagent Grade, 99%), glycine (BioUltra Grade, 99%), N, N-dimethylformamide, 3-aminopropyltriethoxysilane (ACS grade, 99.8%), Tris (ACS grade, 99.9%), potassium hydroxide (Reagent Grade, 99.9%), and trypsin (Proteomics Grade, 95%) were acquired from Sigma-Aldrich (USA). Nitric acid (Reagent Grade, 99.7%) was purchased from Shanghai Chemical Reagent (China). Hydrogen peroxide (Reagent Grade, 99.7%) and tetrandrine hydrochloride (Reagent Grade, 99.7%) were from Yuanye (China). 5/6-Carboxyfluorescein succinimidyl ester was purchased from Thermo-Fisher Scientific (Biological Reagent Grade, 90%). Oxytocin (Chromatography Grade, 97%) was acquired from Macklin (China). MBQ-167 was purchased from MCE (United States Pharmacopeia Grade, 99.86%). SiO_2_@Au was kindly donated by Huan Meng. SiO_2_ with different size and surface charges were synthesized by Wei Huang according to a literature report (56).

### Human Breast Milk Collection.

Milk samples were collected from 53 nursing mothers, aged 21 to 36 y with body mass indexes ranging from 16 to 32, at lactation periods within 1 y. We acquired the consent from all participants. Participants were in Suzhou (39 participants), Zaozhuang (11 participants), and Bozhou (3 participants). Participants were selected based on the following inclusion criteria: i) Participants were healthy breastfeeding mothers aged 20 to 40 y; ii) Participants were within 0 to 12 mo postpartum at the time of milk collection. Additionally, we applied three exclusion criteria to ensure the integrity of the study: i) Participants who reported smoking, taking medications, or using drugs within the 2 wk prior to sample collection; ii) Participants with known exposure to high levels of environmental pollutants (e.g., occupational exposure to heavy metals or nanoparticles) within the past 3 y were excluded; iii) Participants who had experienced mastitis within the 2 wk prior to sample collection were excluded. To prevent contamination, all samples were collected manually following standard guidance (49). The collection procedure involved one hand cupping the breast while the other hand formed a C-shape with the thumb and forefinger approximately 3 to 4 cm below the nipple. Gentle compression and pressure were applied using the thumb and forefinger, followed by a release. This sequence of compression, squeezing, and release was repeated until 100 mL of milk was collected. All samples were stored in uniform milk storage bags. After collection, each sample was labeled, tightly sealed, and stored in the dark at −80 °C until analysis.

### Isolation of NPs from Breast Milk.

To prevent external contamination, all samples were processed in a laminar flow clean bench within a superclean room. Ultrapure solvents were used throughout the experiment, and all equipment and containers were rinsed five times with deionized (DI) water before use. Each milk sample (50 mL) was centrifuged at 45,000 rpm and 4 °C for 50 min using an ultraspeed centrifuge (Optima L-100XP, Beckman, USA) to collect the pellets. To remove biological debris, the pellet was suspended in 10 mL of protease solution (1 mg/mL trypsin) and subjected to water-bath sonication at 150 W for 10 min, followed by incubation at 37 °C for 24 h. The suspension was then centrifuged to collect the pellet, which was subsequently resuspended in 50 mM Tris-HCl buffer (pH 8.2) containing 8 M urea to dissolve residual biological components. The pellet was collected by ultracentrifugation and thoroughly washed three times in 10 mL Tris-HCl buffer and three times in DI water. Finally, the pellet was resuspended in 1 mL DI water for further analysis.

Moreover, an EBL and 53 PBLs were prepared to detect potential NP contamination from the laboratory environment and external sources, respectively. For the EBL, 50 mL of DI water was placed in an uncovered Petri dish and positioned at the experimental site throughout the procedure. For the PBL, 50 mL of particle-free human breast milk (prepared by centrifugation at 45,000 rpm for 50 min) was included and processed using the same procedure as the samples.

### Characterization of Isolated NPs.

The isolated NP suspensions were characterized using TEM, light microscopy, NTA, EDX, XRD, Zeta potential analysis, and ICP-MS. Prior to analysis, all NP suspensions were dispersed by water-bath sonication at 150 W for 10 min. For TEM observations, 5 μL of NP suspension was dropped onto TEM grids and allowed to dry at room temperature. The morphology and elemental compositions of NPs were examined using a TEM system coupled with EDX (Tecnai G2 spirit BioTwin, FEI, USA) at 120 kV. The detection limit of EDX is 1 at% for F and O and 0.1 at% for Si, Fe, Cu, Al, Mg, and Zn. For light microscopy, 20 μL of NP suspension was dropped onto a glass slide, dried at room temperature, and examined using an optical microscope (CKX3-SLP, OLYMPUS, Japan). An aliquot of 700 μL of NP suspension was analyzed by a Nanosight NS300 Nanoparticle Tracking Analyzer (Malvern, UK) to assess particle count and size distribution. The detection limit of NTA is 1 × 10^6^ particles/mL. A Zetasizer Nano instrument (Zetasizer Nano ZS90, Malvern, UK) was used to determine the Zeta potential of the NPs. For ICP-MS analysis, 500 μL aliquots of NP suspensions were digested in 1 mL of 65 to 70% nitric acid (HNO_3_) with 0.5 mL of hydrogen peroxide, heated at 120 °C for 3 h, and then diluted to a final volume of 5 mL with DI water. The detection limit of ICP-MS is 50 ppt for Si, 1 ppt for Fe, Cu, Al, Mg, and Zn and 100 ppt for Ti. To determine the crystalline form of the NPs, 10 mL aliquots from human breast milk samples were pooled, and NPs were isolated following the protocol above. The NP samples were dried at −80 °C for 48 h and analyzed using an X-ray powder diffractometer (ESCALAB 250XI, Thermo Fisher Scientific, USA).

### Preparation of FITC-Labeled SiO_2_ NPs.

SiO_2_ NPs were labeled with FITC for biodistribution analysis according to a reported method (57). Specifically, 400 μL of 3-aminopropyltriethoxysilane was added to a suspension of SiO_2_ NPs (5 mg) in 10 mL of anhydrous, oxygen-free N, N-dimethylformamide (DMF), and the mixture was stirred at room temperature for 24 h. The suspension was then centrifuged at 16,000 rpm for 10 min to collect the SiO_2_ NP pellet. The pellet was resuspended in 5 mL of DI water containing 1 mg/mL of FITC-NHS and reacted under stirring at room temperature for an additional 24 h. The resulting FITC-labeled SiO_2_ NPs were centrifuged, washed three times with DI water, and stored in 1 mL of DI water for further use.

### Animal Treatment.

Eight-week-old ICR mice were obtained from Cavens Biological Technology (Changzhou, Jiangsu, China) and housed in a specific-pathogen-free (SPF) environment with controlled conditions (temperature: 20 to 26 °C, humidity: 40 to 70%, 12-h light/dark cycle). Males and females were housed separately for 1 wk prior to pairing to synchronize reproductive cycles. For mating, three females were placed with one male in a single cage and left together for 2 to 3 d to allow copulation. Vaginal plugs were checked each morning to confirm successful mating; upon detection of a plug, females were separated and housed individually with nesting material. Pregnancy was monitored through physical and behavioral indicators such as weight gain and nesting activity. After parturition, mothers and pups were left undisturbed for 48 h to ensure maternal bonding, and pups were weaned at 4 wk of age. In this study, 64 female mice were used, including 24 mice in milk quantification analysis, 25 mice in structure–activity relationship (SAR) analysis, and 15 mice in transport pathway analysis.

Female mice were exposed to SiO_2_ NPs via oropharyngeal aspiration (OPA) or intragastric (IG) administration according to established protocols (58, 59). It has been estimated that the intake of nanosilica from food products ranges from 0.3 to 33 mg nanosilica/day for an adult (60). Based on this estimation, we converted the human exposure dose to an equivalent dose for mice, resulting in a range of 0.43 to 47.46 mg/kg body weight/week of SiO_2_ NPs (61). Additionally, previous studies have reported that silica concentrations in nonoccupational dust exposure can range from 0.15 to 5.75 mg/m^3^ (62). Using established methods for calculating aspiration doses in mice based on human exposure levels (63), we derived a final dose range of 0.18 to 6.82 mg/kg body weight/week for NPs administered via oropharyngeal aspiration (OPA). Based on these calculations, the animals were administered 2 mg/kg SiO_2_ in seven exposure sessions throughout the 7-wk study. The SiO_2_ NP samples were dispersed in PBS (1 mg/mL) by water-bath sonication before exposure. The mice were divided into nine groups: Group 1: 2 mg/kg of FITC-SiO_2_@Au by OPA; Group 2: 2 mg/kg of FITC-SiO_2_@Au by IG; Group 3: 2 mg/kg of FITC-SiO_2_-ma by IG; Group 4: 2 mg/kg of FITC-SiO_2_-la by IG; Group 5: 2 mg/kg of FITC-SiO_2_-sa by IG; Group 6: 2 mg/kg of FITC-SiO_2_-sc by IG; Group 7: 2 mg/kg of FITC-SiO_2_-sn by IG; Group 8: vehicle solution by OPA; and Group 9: vehicle solution by IG. Each animal received NP exposure seven times. As outlined in Fig. 3*B*, females received two exposures to SiO_2_ NPs prior to cohabitation with males. During pregnancy and lactation, animals were exposed to SiO_2_ NPs on days 13, 19, 25, 34 and 40. Following the protocol reported by DePeters et al. (53), milk samples were collected daily from lactating mice beginning on the third day postpartum and continuing for 18 d. All mouse milk samples were stored at −80 °C for further analysis.

### CE-LIF Analysis.

A capillary electrophoresis (CE) system equipped with a laser-induced fluorescence detector and an Argon ion laser source was developed to detect FITC-labeled SiO_2_ NPs in mouse breast milk samples and standards. Standards were prepared by diluting FITC-labeled SiO_2_ NPs in mouse milk samples from vehicle control groups. The separation was performed using a bare fused silica capillary with an internal diameter of 100 μm and a total length of 100 cm. Milk samples were injected into the capillary at a pressure of 2 psi for 10 s. The separation was carried out at a pressure of 6 psi using 25 mM Tris plus 192 mM Glycine (TG) buffer (pH 8.3). Between runs, the capillary was flushed at 5 psi with 0.1 M NaOH for 3 min, followed by water for 2 min, and then TG buffer for 3 min to ensure optimal separation conditions. The detection limit of CE-LIF for FITC-labeled SiO_2_ NPs is 10 ng/mL.

### Intravital Microscopy Imaging.

Lactating mice were prepared for intravital microscopy imaging by shaving the abdominal area. ISI and TI mouse models were established using pharmacological interventions according to literature reports (64, 65). To establish the ISI model, SIN was dissolved in PBS solution at a concentration of 5 mg/mL. The lactating mice were injected intraperitoneally with 10 mg/kg SIN 24 h before SiO_2_ NP exposure. For TI establishment, MBQ-167 was dissolved in a mixture solution of DMSO/PEG 300/Tween-80/Saline (2/8/1/9) at 2.5 mg/mL. The lactating mice were injected intraperitoneally with 10 mg/kg MBQ-167 1 h before SiO_2_ NP exposure. All injections were performed using a 1 mL insulin syringe with a 29G needle, ensuring precise dosing and minimal stress to the animals. All injections were performed using a 1 mL insulin syringe with a 29G needle, ensuring precise dosing and minimal stress to the animals. Mice were then exposed to 14 mg/kg FITC-SiO_2_ via IG administration. The anesthetized mice underwent a surgical procedure to create a 10 mm diagonal incision at the top of the fourth mammary fat pad, using dissection scissors to expose the mammary tissue. An imaging slide was placed over the exposed mammary tissue, and the mice were secured under a live imaging microscope (IVM-C3, IVIM Technology, Korea) to identify mammary blood vessel positions and record fluorescence signals. For vascular contrast, 100 μL of 1.5% Evans Blue dye was injected into the tail vein. Throughout imaging, mice were sedated with 1.5 to 2% (v/v) isoflurane in oxygen delivered via inhalation anesthesia and placed in a custom-designed imaging box. Mice were kept under anesthesia on a heating pad maintained at 37 °C to ensure stable body temperature during the imaging process.

### Statistical Analysis.

All animals were randomly assigned to each experimental group using a randomization method (e.g., throwing dice) to ensure unbiased group allocation. The results are presented as the mean ± SD of at least three replicates. Statistical analyses were performed using IBM SPSS Statistics 25. Multivariate linear regression analysis was conducted to assess the relationships between the concentration of NPs in human breast milk and potential influencing factors. The multivariate model included all potential factors as covariates, and the enter method was used for model selection. For comparisons among multiple groups, one-way ANOVA was performed, followed by post hoc Tukey HSD tests to identify specific group differences. A significance level of *P* < 0.05 was used to determine statistical significance.

## Supplementary Material

Appendix 01 (PDF)

Movie S1. **Distribution of FITC-SiO_2_ in mouse blood vessels**. Real-time visualization of FITC-SiO_2_ NPs within mouse blood vessels, captured using intravital microscopy. BV indicates blood vessel.

Movie S2. **Distribution of FITC-SiO_2_ in mouse mammary glands**. Real-time visualization of FITC-SiO_2_ NPs within mouse mammary glands, captured using intravital microscopy. AL indicates alveolar lumen.

## Data Availability

The data have been deposited in the Harvard Dataverse Repository (66).

## References

[1] D. Xu , Complement in breast milk modifies offspring gut microbiota to promote infant health. Cell **187**, 750–763 (2024). 38242132 10.1016/j.cell.2023.12.019 PMC10872564

[2] A. Paredes , γ-Linolenic acid in maternal milk drives cardiac metabolic maturation. Nature **618**, 365–373 (2023). 37225978 10.1038/s41586-023-06068-7

[3] C. J. Stewart, Diet-microbe-host interaction in early life. Science **381**, 38–38 (2023). 10.1126/science.adi6318 37410822

[4] R. Pérez-Escamilla , Breastfeeding: Crucially important, but increasingly challenged in a market-driven world. Lancet **401**, 472–485 (2023). 36764313 10.1016/S0140-6736(22)01932-8

[5] G. Yu , Healthy dietary patterns are associated with exposure to environmental chemicals in a pregnancy cohort. Nat. Food **5**, 563–568 (2024). 38951691 10.1038/s43016-024-01013-x PMC11272572

[6] G. M. Lehmann , Environmental chemicals in breast milk and formula: Exposure and risk assessment implications. Environ. Health Perspect. **126**, 096001 (2018). 30187772 10.1289/EHP1953 PMC6375394

[7] J. A. Goodrich, H. Hampson, Complex interplay of diet and chemical exposures during pregnancy. Nat. Food **5**, 646–647 (2024). 39095654 10.1038/s43016-024-01026-6

[8] J. M. Braun, Early-life exposure to EDCs: Role in childhood obesity and neurodevelopment. Nat. Rev. Endocrinol. **13**, 161–173 (2017). 27857130 10.1038/nrendo.2016.186 PMC5322271

[9] World Health Organization. Guidance for Identifying Populations at Risk from Mercury Exposure. United Nations Environment Programme, World Health Organization (2008). https://www.who.int/publications/m/item/guidance-for-identifying-populations-at-risk-from-mercury-exposure. Accessed 15 March 2025.

[10] F. Fish, What pregnant woman and parents should know (Draft updated advice US Food and Drug Administration, US Department of Health and Human Services, 2014).

[11] Centers for Disease Control and Prevention, Guidelines for the identification and management of lead exposure in pregnant and lactating women, U.S. Department of Health and Human Services, Centers for Disease Control and Prevention (2010). https://stacks.cdc.gov/view/cdc/147837. Accessed 16 March 2025.

[12] USFDA, FDA Strategy for Monitoring, Method Development, and Reducing Human Exposure to Dioxins (US Food and Drug Administration, 2002), https://www.fda.gov/food/environmental-contaminants-food/fda-strategy-monitoring-method-development-and-reducing-human-exposure-dioxins.

[13] USFDA, FDA Announces PFAS Used in Grease-Proofing Agents for Food Packaging No Longer Being Sold in the U.S. (US Food and Drug Administration, 2024), https://www.fda.gov/food/hfp-constituent-updates/fda-announces-pfas-used-grease-proofing-agents-food-packaging-no-longer-being-sold-us?eType=EmailBlastContent&eId=d3f89efb-fb3d-42df-a6d0-1dbb6cdf6dbc.

[14] S. S. Selph , Screening for HIV infection in pregnant women: Updated evidence report and systematic review for the US Preventive Services Task Force. JAMA **321**, 2349–2360 (2019). 31184704 10.1001/jama.2019.2593

[15] N. A. Terrault, M. T. Levy, K. W. Cheung, G. Jourdain, Viral hepatitis and pregnancy. Nat. Rev. Gastroenterol. Hepatol. **18**, 117–130 (2021). 33046891 10.1038/s41575-020-00361-w

[16] T. Jamnik , Next-generation biomonitoring of the early-life chemical exposome in neonatal and infant development. Nat. Commun. **13**, 2653 (2022). 35550507 10.1038/s41467-022-30204-y PMC9098442

[17] U.S. Food and Drug Administration and U.S. Environmental Protection Agency. Advice about eating fish for those who might become or are pregnant or breastfeeding and children Ages 1–11 years. U.S. Food and Drug Administration (2021). https://www.fda.gov/media/102331/download?attachment. Accessed 15 March 2025.

[18] A. Witczak , Changes in polychlorinated biphenyl residues in milk during lactation: Levels of contamination, influencing factors, and infant risk assessment. Int. J. Mol. Sci. **23**, 12717 (2022). 36361507 10.3390/ijms232112717 PMC9655485

[19] World Health Organization. Guideline: updates on HIV and infant feeding: the duration of breastfeeding, and support from health services to improve feeding practices among mothers living with HIV. World Health Organization (2016). https://iris.who.int/bitstream/handle/10665/246260/9789241549707-eng.pdf?sequence=1. Accessed 15 March 2025. 27583316

[20] K. Yamashita , Silica and titanium dioxide nanoparticles cause pregnancy complications in mice. Nat. Nanotechnol. **6**, 321–328 (2011). 21460826 10.1038/nnano.2011.41

[21] J. Cai , Translocation of transition metal oxide nanoparticles to breast milk and offspring: The necessity of bridging mother-offspring-integration toxicological assessments. Environ. Int. **133**, 105153 (2019). 31520958 10.1016/j.envint.2019.105153

[22] Y. Morishita , Distribution of silver nanoparticles to breast milk and their biological effects on breast-fed offspring mice. ACS Nano **10**, 8180–8191 (2016). 27498759 10.1021/acsnano.6b01782

[23] C. Svendsen , Key principles and operational practices for improved nanotechnology environmental exposure assessment. Nat. Nanotechnol. **15**, 731–742 (2020). 32807878 10.1038/s41565-020-0742-1

[24] X. Cai , Molecular mechanisms, characterization methods, and utilities of nanoparticle biotransformation in nanosafety assessments. Small **16**, 1907663 (2020). 10.1002/smll.201907663 32406193

[25] J. P. Ryman-Rasmussen , Inhaled carbon nanotubes reach the subpleural tissue in mice. Nat. Nanotechnol. **4**, 747–751 (2009). 19893520 10.1038/nnano.2009.305 PMC2783215

[26] W. Hill , Lung adenocarcinoma promotion by air pollutants. Nature **616**, 159–167 (2023). 37020004 10.1038/s41586-023-05874-3 PMC7614604

[27] B. B. Ural , Inhaled particulate accumulation with age impairs immune function and architecture in human lung lymph nodes. Nat. Med. **28**, 2622–2632 (2022). 36411343 10.1038/s41591-022-02073-x PMC9835154

[28] J. R. Turner, Intestinal mucosal barrier function in health and disease. Nat. Rev. Immunol. **9**, 799–809 (2009). 19855405 10.1038/nri2653

[29] S. Ganesan, A. T. Comstock, U. S. Sajjan, Barrier function of airway tract epithelium. Tissue Barriers **1**, e24997 (2013). 24665407 10.4161/tisb.24997 PMC3783221

[30] F. M. Hannan , Hormonal regulation of mammary gland development and lactation. Nat. Rev. Endocrinol. **19**, 46–61 (2023). 36192506 10.1038/s41574-022-00742-y

[31] A. C. Rios , Essential role for a novel population of binucleated mammary epithelial cells in lactation. Nat. Commun. **7**, 11400 (2016). 27102712 10.1038/ncomms11400 PMC4844753

[32] O. T. Oftedal, The evolution of lactation in mammalian species. Milk Mucosal Immun. Microbiome Impact Neonate **94**, 1–10 (2020). 10.1159/000505577 32155641

[33] O. Wellnitz, R. Bruckmaier, Invited review: The role of the blood–milk barrier and its manipulation for the efficacy of the mammary immune response and milk production. J. Dairy Sci. **104**, 6376–6388 (2021). 33773785 10.3168/jds.2020-20029

[34] A. J. Prendergast , Transmission of CMV, HTLV-1, and HIV through breastmilk. Lancet Child Adolesc. Health **3**, 264–273 (2019). 30878119 10.1016/S2352-4642(19)30024-0

[35] C. Atyeo, G. Alter, The multifaceted roles of breast milk antibodies. Cell **184**, 1486–1499 (2021). 33740451 10.1016/j.cell.2021.02.031

[36] Z. Wang , Effects of silver nanoparticles on maternal mammary glands and offspring development under lactation exposure. Ecotoxicol. Environ. Saf. **256**, 114869 (2023). 37037110 10.1016/j.ecoenv.2023.114869

[37] C. Zhang , Induction of size-dependent breakdown of blood-milk barrier in lactating mice by TiO2 nanoparticles. PLoS One **10**, e0122591 (2015). 25849145 10.1371/journal.pone.0122591 PMC4388820

[38] J. Wu , Dual effects of JNK activation in blood-milk barrier damage induced by zinc oxide nanoparticles. J. Hazard. Mater. **399**, 122809 (2020). 32937690 10.1016/j.jhazmat.2020.122809

[39] J. Wang , Exposure to ZnO nanoparticles induced blood-milk barrier dysfunction by disrupting tight junctions and cell injury. Toxicol. Lett. **384**, 63–72 (2023). 37437672 10.1016/j.toxlet.2023.07.004

[40] A. Hussain , Postnatal distribution of ZnO nanoparticles to the breast milk through oral route and their risk assessment for breastfed rat offsprings. Hum. Exp. Toxicol. **39**, 1318–1332 (2020). 32347117 10.1177/0960327120921441

[41] L. Yao , Toxic effects of TiO2 NPs in the blood-milk barrier of the maternal dams and growth of offspring. Ecotoxicol. Environ. Saf. **208**, 111762 (2021). 33396082 10.1016/j.ecoenv.2020.111762

[42] A. Nel , Toxic potential of materials at the nanolevel. Science **311**, 622–627 (2006). 16456071 10.1126/science.1114397

[43] M. Heringa , Detection of titanium particles in human liver and spleen and possible health implications. Part. Fibre Toxicol. **15**, 1–9 (2018). 29642936 10.1186/s12989-018-0251-7 PMC5896156

[44] R. J. Peters , Silicon dioxide and titanium dioxide particles found in human tissues. Nanotoxicology **14**, 420–432 (2020). 31994971 10.1080/17435390.2020.1718232

[45] Y. Qi , Intrusion of inhaled exotic ultrafine particles into the knee joint in humans and animals: A risk to the joint and surrounding tissues. Nano Today **43**, 101426 (2022).

[46] H. Bové , Ambient black carbon particles reach the fetal side of human placenta. Nat. Commun. **10**, 3866 (2019). 31530803 10.1038/s41467-019-11654-3 PMC6748955

[47] J. Kolosnjaj-Tabi , Anthropogenic carbon nanotubes found in the airways of Parisian children. EBioMedicine **2**, 1697–1704 (2015). 27014740 10.1016/j.ebiom.2015.10.012 PMC4793559

[48] S. F. Hansen, A. Lennquist, Carbon nanotubes added to the SIN List as a nanomaterial of Very High Concern. Nat. Nanotechnol. **15**, 3–4 (2020). 31925393 10.1038/s41565-019-0613-9

[49] Y. Grosse , Carcinogenicity of fluoro-edenite, silicon carbide fibres and whiskers, and carbon nanotubes. Lancet Oncol. **15**, 1427–1428 (2014). 25499275 10.1016/S1470-2045(14)71109-X

[50] C. T. Migliaccio , Respiratory and systemic impacts following MWCNT inhalation in B6C3F1/N mice. Part. Fibre Toxicol. **18**, 1–21 (2021). 33771183 10.1186/s12989-021-00408-z PMC7995731

[51] World Health Organization, WHO global air quality guidelines: Particulate matter (PM2.5 and PM10), ozone, nitrogen dioxide, sulfur dioxide and carbon monoxide (2021), https://iris.who.int/handle/10665/345329. 34662007

[52] A. Ragusa , Raman microspectroscopy detection and characterisation of microplastics in human breastmilk. Polymers **14**, 2700 (2022). 35808745 10.3390/polym14132700 PMC9269371

[53] C. Cosemans , Black carbon particles in human breast milk: Assessing infant’s exposure. Front. Public Health **11**, 1333969 (2024). 38298262 10.3389/fpubh.2023.1333969 PMC10828029

[54] A. M. Gatti , Heavy metal nanoparticle detection in human and formula milk. Foods **13**, 3178 (2024). 39410213 10.3390/foods13193178 PMC11475992

[55] J. Cai , Mammary leukocyte-assisted nanoparticle transport enhances targeted milk trace mineral delivery. Adv. Sci. **9**, 2200841 (2022). 10.1002/advs.202200841 PMC9475556 35773238

[56] X. Chen , Matrix-induced defects and molecular doping in the afterglow of SiO2 microparticles. Nat. Commun. **15**, 8111 (2024). 39285162 10.1038/s41467-024-51591-4 PMC11405531

[57] X. Liu , Doped graphene to mimic the bacterial NADH oxidase for one-step NAD+ supplementation in mammals. J. Am. Chem. Soc. **145**, 3108–3120 (2023). 36700857 10.1021/jacs.2c12336

[58] M. Gao , Nano-microflora interaction inducing pulmonary inflammation by pyroptosis. Environ. Sci. Technol. **58**, 8643–8653 (2024). 38676641 10.1021/acs.est.4c00141

[59] R. Wang , Treatment of peanut allergy and colitis in mice via the intestinal release of butyrate from polymeric micelles. Nat. Biomed. Eng. **7**, 38–55 (2023). 36550307 10.1038/s41551-022-00972-5 PMC9870785

[60] S. Dekkers , Presence and risks of nanosilica in food products. Nanotoxicology **5**, 393–405 (2011). 20868236 10.3109/17435390.2010.519836

[61] S. Reagan-Shaw , Dose translation from animal to human studies revisited. FASEB J. **22**, 659–661 (2008). 17942826 10.1096/fj.07-9574LSF

[62] X. Xu , A study of siliceous pneumoconiosis in a desert area of Sunan County, Gansu Province, China. Biomed. Environ. Sci. **6**, 217–222 (1993). 8292266

[63] J. Cao , Deciphering key nano-bio interface descriptors to predict nanoparticle-induced lung fibrosis. Part. Fibre Toxicol. **22**, 1 (2025). 39810232 10.1186/s12989-024-00616-3 PMC11731361

[64] W.-J. Gao , Suppression of macrophage migration by down-regulating Src/FAK/P130Cas activation contributed to the anti-inflammatory activity of sinomenine. Pharmacol. Res. **167**, 105513 (2021). 33617975 10.1016/j.phrs.2021.105513

[65] J. Reig-López , Physiologically-based pharmacokinetic/pharmacodynamic model of MBQ-167 to predict tumor growth inhibition in mice. Pharmaceutics **12**, 975 (2020). 33076517 10.3390/pharmaceutics12100975 PMC7602742

[66] Q. Yang, Data from “Identification of nanoparticle infiltration in human breast milk: Chemical profiles and trajectory pathways.” Harvard Dataverse. 10.7910/DVN/IS5FKX. Deposited 22 April 2025. PMC12107167 40354532

