# Supplementary material for: Identification of nanoparticle infiltration in human breast milk: Chemical profiles and trajectory pathways
Source: Proc Natl Acad Sci U S A. 2025 May 12;122(20):e2500552122. doi: 10.1073/pnas.2500552122 (PMC12107167; doi:10.1073/pnas.2500552122)
Supplement: Supplementary file 1 — Appendix 01 (PDF) [file pnas.2500552122.sapp.pdf]

Supplementary Tables

Table S1 Basic information, air particulate exposure, dietary intake and use of possible NP-containing products reported by the 53 nursing mothers

Sample ID	Age (year)	Body mass index	Lactation (month)	PM2.5 (μg/m^3)[a]	Household spray use	Printer use	Daily water intake	Weekly flour intake	Weekly solid beverages intake	Weekly seafood intake	Weekly seasoning powder intake	Whitening toothpaste use	Weekly sunscreen use	Weekly makeup use
1	30	24.68	3	26	No	Yes	>1000 mL	>2 times	≤1time	≤1time	≤1time	No	>2 times	≤1time
2	28	22.03	3	26	No	Yes	>1000 mL	≤1time	≤1time	>2 times	≤1time	No	>2 times	≤1time
3	26	23.44	2	88	No	No	>1000 mL	>2 times	≤1time	≤1time	>2 times	Yes	≤1time	≤1time
4	31	22.89	7	55	No	Yes	>1000 mL	>2 times	≤1time	≤1time	>2 times	No	>2 times	≤1time
5	31	22.58	3	26	No	No	>1000 mL	≤1time	≤1time	>2 times	≤1time	No	>2 times	>2 times
6	36	21.76	5	40	No	Yes	≤1000 mL	>2 times	≤1time	>2 times	≤1time	No	≤1time	≤1time
7	28	21.94	3	40	No	No	>1000 mL	>2 times	≤1time	≤1time	≤1time	No	≤1time	≤1time
8	31	20.40	3	19	No	Yes	>1000 mL	≤1time	>2 times	≤1time	>2 times	No	≤1time	≤1time
9	31	20.83	9	36	No	Yes	>1000 mL	≤1time	>2 times	≤1time	≤1time	No	>2 times	≤1time
10	28	19.28	7	18	No	Yes	>1000 mL	≤1time	≤1time	>2 times	≤1time	No	≤1time	≤1time
11	26	20.45	4	21	No	No	≤1000 mL	>2 times	≤1time	≤1time	>2 times	No	>2 times	≤1time
12	35	22.66	4	23	No	Yes	≤1000 mL	≤1time	≤1time	≤1time	≤1time	No	≤1time	≤1time
13	27	21.05	3	21	No	No	>1000 mL	>2 times	>2 times	≤1time	≤1time	No	≤1time	≤1time
14	30	20.55	4	19	No	No	≤1000 mL	>2 times	≤1time	>2 times	>2 times	No	≤1time	≤1time
15	33	30.10	3	19	No	No	>1000 mL	>2 times	≤1time	>2 times	≤1time	No	≤1time	≤1time
16	33	19.05	11	43	Yes	No	≤1000 mL	≤1time	≤1time	≤1time	≤1time	No	>2 times	>2 times
17	25	16.42	6	36	No	No	>1000 mL	≤1time	>2 times	>2 times	>2 times	No	>2 times	>2 times
18	35	29.52	10	43	No	No	>1000 mL	≤1time	≤1time	≤1time	>2 times	No	≤1time	≤1time
19	31	17.22	6	20	No	No	>1000 mL	>2 times	≤1time	≤1time	≤1time	No	≤1time	≤1time
20	35	21.48	3	18	No	Yes	≤1000 mL	≤1time	≤1time	≤1time	≤1time	No	≤1time	≤1time
21	26	23.44	4	43	Yes	No	>1000 mL	≤1time	≤1time	≤1time	>2 times	Yes	>2 times	>2 times
22	27	21.26	3	43	No	Yes	>1000 mL	≤1time	≤1time	≤1time	≤1time	No	>2 times	>2 times
23	31	19.68	4	18	No	No	>1000 mL	≤1time	≤1time	>2 times	≤1time	No	>2 times	≤1time
24	21	31.46	5	43	Yes	No	≤1000 mL	>2 times	≤1time	>2 times	>2 times	No	≤1time	≤1time
25	24	21.64	6	22	No	No	>1000 mL	≤1time	≤1time	>2 times	≤1time	No	>2 times	≤1time

26	28	25.39	3	27	No	No	>1000 mL	≤1time	≤1time	≤1time	≤1time	Yes	>2 times	>2 times
27	21	23.03	5	37	Yes	No	>1000 mL	>2 times	≤1time	>2 times	≤1time	No	>2 times	≤1time
28	24	21.26	5	42	Yes	No	>1000 mL	>2 times	>2 times	>2 times	>2 times	Yes	≤1time	≤1time
29	26	16.69	7	20	Yes	No	>1000 mL	≤1time	≤1time	>2 times	≤1time	No	≤1time	≤1time
30	36	27.64	5	37	Yes	No	>1000 mL	>2 times	≤1time	≤1time	>2 times	Yes	≤1time	≤1time
31	23	25.32	3	67	No	No	≤1000 mL	≤1time	≤1time	>2 times	>2 times	No	≤1time	≤1time
32	35	25.00	1	18	No	No	≤1000 mL	≤1time	>2 times	≤1time	≤1time	No	≤1time	≤1time
33	28	23.44	7	20	No	No	≤1000 mL	≤1time	≤1time	≤1time	≤1time	Yes	≤1time	≤1time
34	29	21.63	4	55	No	Yes	≤1000 mL	>2 times	>2 times	>2 times	≤1time	No	>2 times	>2 times
35	28	21.47	6	47	Yes	Yes	≤1000 mL	>2 times	≤1time	≤1time	>2 times	Yes	≤1time	≤1time
36	30	22.15	4	18	No	Yes	>1000 mL	≤1time	≤1time	≤1time	≤1time	No	>2 times	≤1time
37	28	18.37	3	23	No	Yes	>1000 mL	≤1time	≤1time	>2 times	≤1time	No	≤1time	≤1time
38	31	20.81	6	18	No	No	>1000 mL	>2 times	≤1time	≤1time	≤1time	No	≤1time	≤1time
39	28	24.46	3	18	No	No	≤1000 mL	>2 times	≤1time	≤1time	>2 times	No	>2 times	≤1time
40	24	24.03	5	20	Yes	Yes	>1000 mL	≤1time	≤1time	≤1time	≤1time	No	≤1time	≤1time
41	29	24.01	3	18	Yes	No	≤1000 mL	>2 times	>2 times	≤1time	≤1time	No	≤1time	≤1time
42	25	21.94	3	55	No	No	≤1000 mL	>2 times	≤1time	>2 times	>2 times	No	≤1time	≤1time
43	28	22.46	4	36	Yes	Yes	>1000 mL	>2 times	≤1time	≤1time	≤1time	No	>2 times	≤1time
44	31	22.68	4	30	Yes	Yes	>1000 mL	≤1time	≤1time	>2 times	≤1time	No	>2 times	≤1time
45	26	21.94	5	27	No	No	≤1000 mL	>2 times	≤1time	≤1time	>2 times	Yes	>2 times	≤1time
46	23	24.98	7	27	No	Yes	>1000 mL	>2 times	≤1time	≤1time	>2 times	No	>2 times	≤1time
47	33	27.34	3	27	Yes	Yes	≤1000 mL	>2 times	≤1time	≤1time	>2 times	Yes	≤1time	≤1time
48	32	21.76	6	30	Yes	Yes	>1000 mL	≤1time	≤1time	>2 times	≤1time	No	>2 times	≤1time
49	26	25.40	9	27	No	Yes	≤1000 mL	≤1time	≤1time	>2 times	≤1time	No	≤1time	≤1time
50	24	21.97	2	27	Yes	No	>1000 mL	≤1time	≤1time	≤1time	>2 times	Yes	>2 times	>2 times
51	23	24.24	6	27	No	No	≤1000 mL	>2 times	≤1time	>2 times	>2 times	No	≤1time	≤1time
52	30	21.94	5	27	Yes	No	>1000 mL	>2 times	>2 times	≤1time	≤1time	No	>2 times	≤1time
53	28	20.83	7	27	No	No	>1000 mL	≤1time	≤1time	≤1time	>2 times	No	≤1time	≤1time

[a] The average daily $PM_{2.5}$ levels in the residential environment of the mothers were measured during the month of sample collection

Table S2 Concentrations of Si and metal elements in NP pellets

Sample ID	Element concentration (ng/mL)						
	Si	Al	Fe	Cu	Ti	Zn	Mg
1	97.96	11.04	12.56	0.82	1.68	23.74	9.62
2	51.70	7.84	11.4	0.66	0.52	18.98	7.98
3	ND	ND	ND	ND	ND	ND	ND
4	ND	ND	ND	ND	ND	ND	ND
5	7.34	24.92	12.06	12.84	14.12	2.74	5.72
6	32.94	10.14	20.96	31.12	6.90	12.16	11.52
7	8.82	4.96	10.06	9.70	8.52	12.06	9.98
8	99.6	11.04	6.56	4.00	12.70	14.96	17.4
9	14.64	10.80	10.76	2.92	4.60	4.84	11.32
10	6.14	17.42	33.60	1.80	1.76	1.72	6.04
11	95.00	8.90	7.92	1.00	0.50	4.5	7.88
12	122.14	3.74	6.16	1.48	2.42	16.34	11.2
13	ND	ND	ND	ND	ND	ND	ND
14	ND	ND	ND	ND	ND	ND	ND
15	121.02	27.36	9.68	1.18	1.92	6.64	9.68
16	ND	ND	ND	ND	ND	ND	ND
17	52.8	9.24	5.12	1.46	0.94	4.16	7.72
18	18.14	9.46	6.54	0.98	0.72	11.46	15.58
19	ND	ND	ND	ND	ND	ND	ND
20	22.40	9.50	8.86	0.98	3.60	2.50	8.58
21	61.00	43.30	3.42	0.48	1.22	0.62	5.98
22	10.60	22.06	177.00	1.10	1.48	11.74	9.54
23	142.66	4.48	9.74	0.68	0.48	3.50	6.84
24	4.94	11.70	10.46	0.94	0.78	6.92	10.38
25	ND	ND	ND	ND	ND	ND	ND
26	ND	ND	ND	ND	ND	ND	ND
27	63.78	3.38	8.88	1.20	0.72	6.06	5.02
28	32.20	8.08	9.20	2.28	0.76	2.96	8.90
29	3.66	12.20	16.48	3.88	4.40	8.26	10.44
30	15.96	13.62	12.48	2.68	0.96	3.56	11.00
31	10.76	28.80	5.60	0.80	1.30	4.50	17.92
32	8.86	22.64	15.96	2.38	0.78	13.52	11.64
33	8.86	32.18	47.72	0.90	0.46	14.64	15.42
34	42.80	6.54	6.50	1.88	0.20	10.96	12.94
35	65.92	3.92	10.36	0.54	0.70	8.04	115.48
36	16.58	11.74	5.42	5.34	1.04	4.3	11.32
37	3.46	15.56	12.08	12.78	2.86	15.94	10.04
38	36.80	10.18	10.54	2.72	0.30	5.78	20.00
39	8.44	17.52	9.44	6.14	0.46	6.66	11.60
40	ND	ND	ND	ND	ND	ND	ND
41	39.14	5.02	11.56	7.74	0.36	6.32	9.82
42	5.46	12.32	10.54	0.62	2.70	5.8	7.06
43	7.36	12.28	11.14	1.74	0.40	3.04	34.66
44	ND	ND	ND	ND	ND	ND	ND
45	10.54	3.30	9.10	1.58	0.42	11.64	13.54
46	15.18	14.16	12.42	0.92	0.64	4.36	8.76
47	14.24	10.84	8.80	0.56	0.32	3.76	45.14
48	57.00	8.12	6.36	1.08	0.48	6.78	69.48
49	ND	ND	ND	ND	ND	ND	ND
50	8.76	3.88	6.82	1.42	0.74	3.48	39.26
51	10.50	17.32	19.48	2.30	0.66	7.58	0.22
52	4.22	14.68	10.46	1.10	1.00	4.02	8.82
53	27.22	12.06	131.80	3.80	0.86	3.66	11.88

Note: ND indicates not detectable.

Table S3 Multivariable linear regression analysis between influencing factors and NP levels in human breast milk

Explanatory variable	Unit of measurement	Unstandardized coefficient		Standardized coefficient Beta	t	P value
		B (10^9)	Std. error (10^9)			
Constant		3.97	9.06		0.438	0.664
PM 2.5 concentration	Number	-1.36	0.22	-0.103	-0.622	0.537
Household spray	Dummy (0=no, 1=yes)	2.33	6.24	0.057	0.373	0.711
Printer use	Dummy (0=no, 1=yes)	-256	6.02	-0.067	-0.425	0.673
Water intake	Dummy (0= less than 1000 ml per day, 1= more than 1000ml per day)	6.84	6.04	0.176	1.131	0.265
Flour intake	Dummy (0=less than 1 time per week, 1= more than 2 times per week)	12.90	6.35	0.346	2.031	**0.049***
Solid beverages intake	Dummy (0= less than 1 time per week, 1= more than 2 times per week)	-4.92	7.32	-0.099	-0.672	0.506
Seafood intake	Dummy (0= less than 1 time per week, 1= more than 2 times per week)	6.72	5.91	0.176	1.138	0.262
Seasoning powder intake	Dummy (0=less than 1 times per week, 1=more than 2 times per week)	-3.79	6.71	-0.100	-0.565	0.575
Whitening toothpaste use	Dummy (0=no, 1=yes)	-1.83	8.66	-0.038	-0.211	0.834
Sunscreen use	Dummy (0= less than 1 time per week, 1= more than 2 times per week)	-2.61	6.59	-0.007	-0.040	0.969
Makeup use	Dummy (0=less than 1 time per week, 1= more than 2 times per week)	2.80	9.97	0.054	0.281	0.780

* Significant, $p<0.05$.

Table S4 Size and surface charge of SiO_2 NPs

SiO_2 NPs	Primary size (nm)	Hydrodynamic size (nm)	Zeta potential (mV)
SiO_2-sa	20	141	-29
SiO_2-ma	50	76	-32.2
SiO_2-la	100	135	-34.8
SiO_2-sc	20	128	19.5
SiO_2-sn	20	156	-8.3

Note: sa, sc, sn, ma, and la refer to SiO_2 properties as follows: sa (small size, anionic charge), sc (small size, cationic charge), sn (small size, neutral charge), ma (medium size, anionic charge), and la (large size, anionic charge).

Supplementary Figures

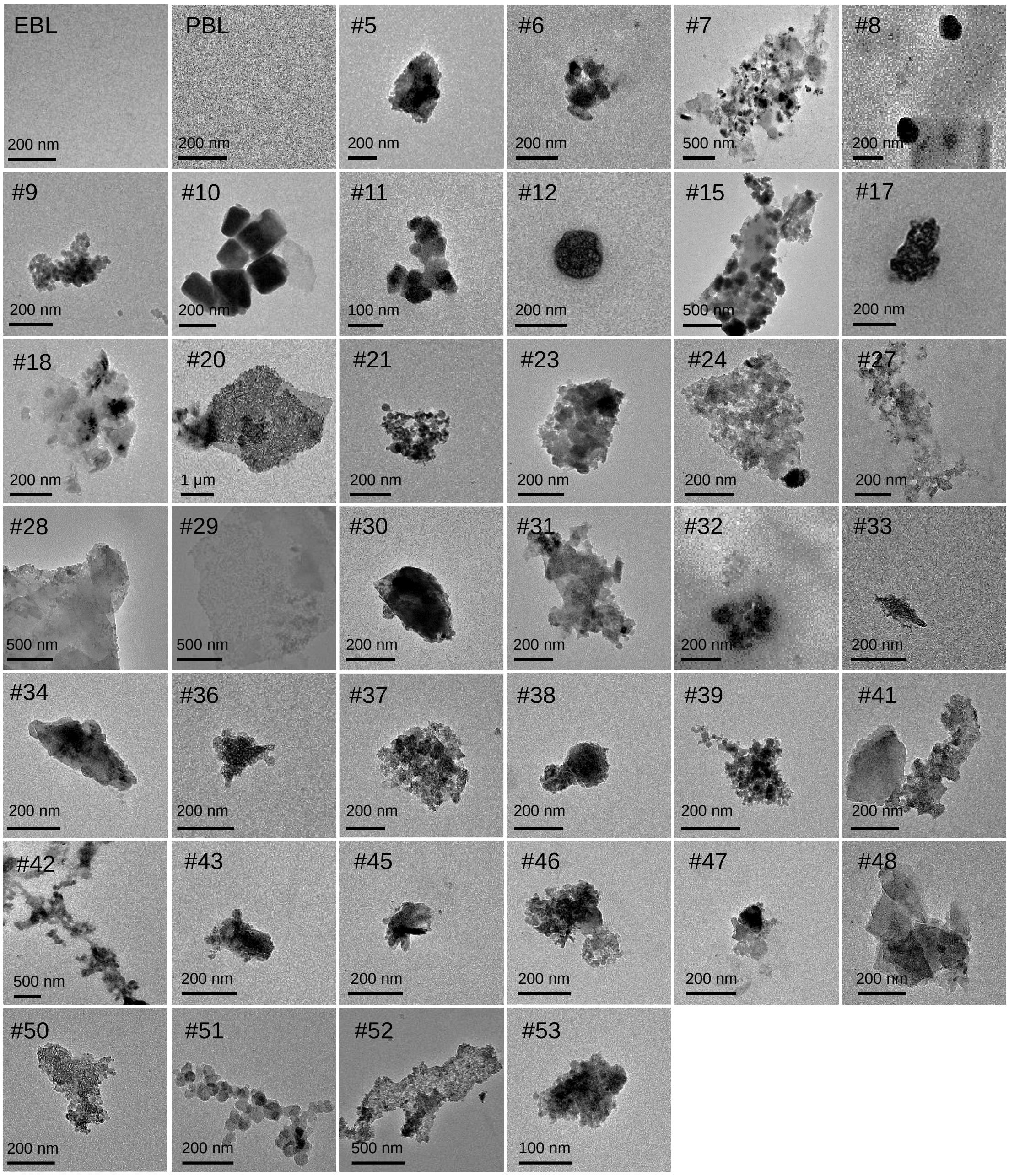

Figure S1 Representative TEM images of NPs isolated from human breast milk

TEM images showing the morphology of NPs isolated from human breast milk samples.

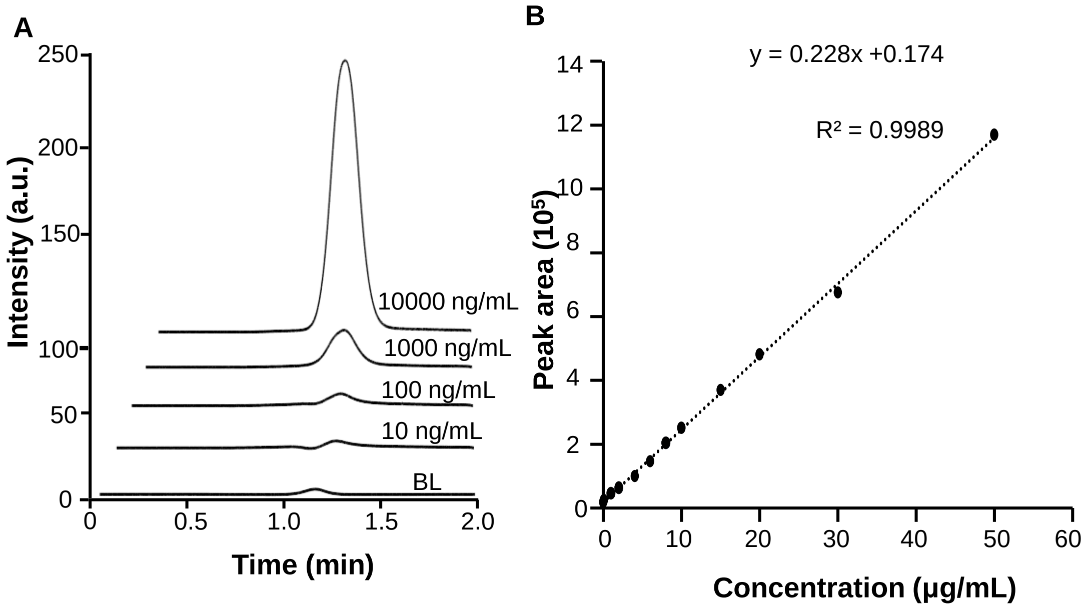

Figure S2 Standard curve of FITC-SiO_2 in breast milk *via* CE-LIF analysis

(A) Representative CE-LIF chromatograms of FITC-SiO_2 standard solutions in breast milk. **(B)** Calibration curve showing peak area *versus* concentration for FITC-SiO_2, with concentrations ranging from 0 to 50 µg/mL. FITC-SiO_2 was diluted in mouse breast milk, and CE-LIF analysis was performed as described in the Methods section.

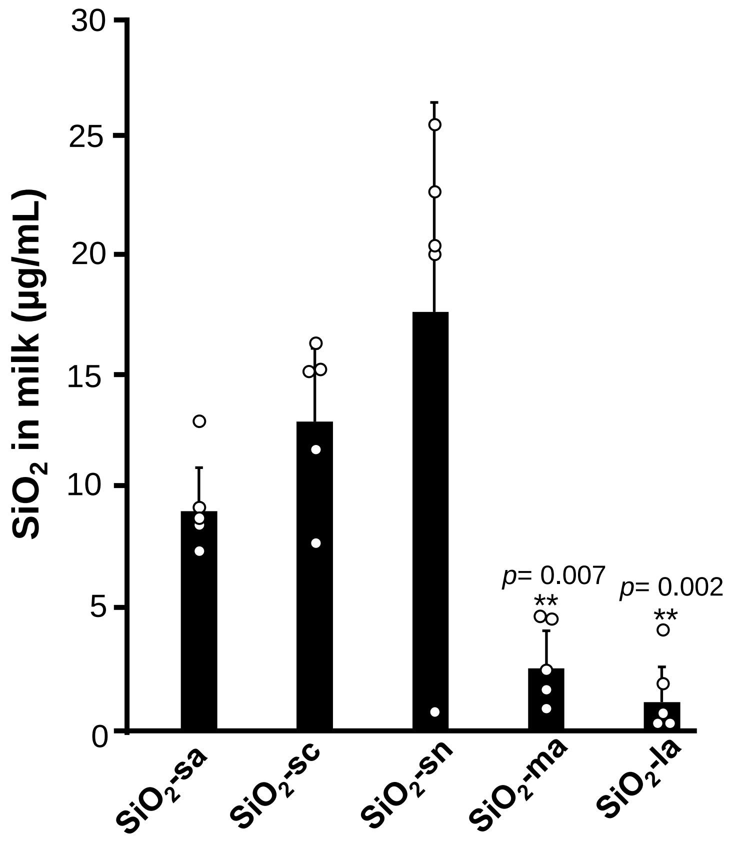

Figure S3 SiO_2 levels in mouse milk following exposure to FITC-SiO_2 with varied sizes and surface charges

Breast milk samples from mice exposed to FITC-labeled SiO_2 NPs with different sizes and surface charges (SiO_2-sa, SiO_2-sc, SiO_2-sn, SiO_2-ma, and SiO_2-la) by IG administration were analyzed by CE-LIF. Five animals were included for each treatment. SiO_2 concentrations were quantified using the standard curve generated from FITC-SiO_2 standards. **$p<0.01$ compared to SiO^2-sa mice by one-way ANOVA.

Supplementary Video

Video S1. Distribution of FITC-SiO_2 in mouse blood vessels

Real-time visualization of FITC-SiO_2 NPs within mouse blood vessels, captured using intravital microscopy. BV indicates blood vessel.

Video S2. Distribution of FITC-SiO_2 in mouse mammary glands

Real-time visualization of FITC-SiO_2 NPs within mouse mammary glands, captured using intravital microscopy. AL indicates alveolar lumen.
